# Warranty*Read before the Keystone Veterinary Medical Association.

**Published:** 1892-05

**Authors:** John S. McKinley


### WARRANTY.*

By The Hon. John S. McKinley.

There is no subject of more interest to us than the one I intend to call your attention to for a few minutes to-night. The fact that there are such strong ties between the two distinct branches of the animal kingdom—man and horse—has caused the horse to be the subject of much consideration by the former, and there is to-day more controversy about the horse than any other animal that man controls or owns ; but the horse becomes a matter of controversy when we consider him as a beast of burden, or as an object of beauty and a means of pleasure, either for riding or driving.

There are but two subjects that men lose their heads about entirely; one is the horse and the other is woman. A man will be reasoned with about politics or religion, but when you come to consider the traits of a horse, there is but one perfect in the whole world, and that is the one he possesses, and strange to say, you never hear anything but the good traits. It has been said by the poet, that "A thing of beauty is a joy forever." I'm sorry to say this does not always apply to the horse, but when you get the beauty and good qualities together, you then have the most attractive and valuable property that life confers, because the value is only regulated by the circumstances of the owner, and hence we find men giving up fabulous sums for the purpose of owning the finest horse.

As the horse is such a source of usefulness as well as pleasure to mankind, he is necessarily the subject of varied and sometimes rapid changes in ownership, and the business of dealing in horses has become simply enormous and almost beyond any comprehension, and for that reason I intend to direct your attention to a few of the well regulated and recognized customs and usages that have grown up among persons buying or selling horses, that have been recognized by law as binding on the parties to the contract. The law relating to the purchase and sale of horses is just like the law relating to any other branch of business; at first crude and sometimes uncertain, it has been made certain and sure by many well-

* Read before the Keystone Veterinary Medical Association.

established principles and decisions of our various courts. If we could only draw on our imagination and go away back into the centuries, how different would we find the law and circumstances surrounding the simple transaction of selling or exchanging a horse.

There would be no flaming advertisements in the public press: "A widow, who has recently lost her husband, and who is about to take a trip to Europe, is desirous of selling her favorite family horse, price no object so the horse gets into the hands of a good, kind master. Apply at stables, rear of No. —— North Broad Street." The anxious buyer would not be met by a woman in deep mourning, accompanied by a servant in full livery, who stands by shedding tears of regret at the thought of parting companionship with an old friend.

Nor would the anxious buyer be scanned from top to bottom and told, "Why, you haven't got money enough to buy this horse." These are but contrivances of the nineteenth century, and by reason of these and many other devices, the law has, to a certain extent, come to the rescue of both the honest seller and honest buyer, and it is sometimes hard to tell which has the more honesty of the two. A horse dealer is something like a lawyer—he seldom tells his adversary all the weak points of his case, and if a horse has any faults he seldom mentions them until pressed very hard. Some fifteen years ago I had made up my mind to purchase a saddle horse. This fact I unfortunately made known to a friend, and by some unfortunate means it came to the ear of a dealer of some notoriety, whom you all know very well should I mention his name, and whom I knew by reputation as having never gotten the worse of a horse deal in his life; he pursued me with wonderful avidity and activity, and while I had no idea of taking advantage of the many good opportunities offered by him to be the owner of a first-class animal, gentle, kind, and at the same time favored by nature with all the gaits, from a running walk to a gentle lope, yet in order to get rid of him I finally agreed to give this fine animal a trial; so, in company with a friend, I called at his stable at an hour appointed, and in a few minutes was mounted on what looked like the animal I was in search of; he worked at first a good deal, like a steam locomotive very hard to get in motion, and the longer I lingered the more he was like the locomotive. After a vigorous application of whip and spur I finally got him out of a walk, and then I found there was no way of getting him into a walk. He paid no attention to anything, but ran up Broad street at full speed; in vain did I apply my full weight and strength to the reins but all to no effect, and after exhausting all my entire strength and all my ingenuity, I was finally brought to a dead halt by his running into an ash-cart and almost breaking my leg. I finally got him turned around and was only too glad to let him take his accustomed snail pace back to the stable. This was my first and only experience with what is known among the trade as a "dummy?" It is unnecessary to say I did not buy the horse. I thanked the owner for his kindness, told him I thought the horse would suit me, but that I would like a day or two to consider the matter.

The law has made an effort to make dealing in horses an honest and honorable business, but with all it is pretty hard to get a man to divulge all the bad qualities of a piece of property he is anxious to dispose of.

The only protection the law affords the purchaser is the guarantee or warranty, which is an agreement and representation made at the time of the sale as to the qualities of the horse; this may be a warranty as to soundness, or kindness, or both. When this contract is in writing, as it should always be, there is seldom much cause for litigation, as the warranty generally expresses the time when the contract or guarantee should cease to exist between the parties; in other words, the vender generally stipulates that the horse should be gentle, kind, sound, fearless of steam, drives single or double, etc., either upon delivery or within a certain time fixed. Of course, where a horse is unkind or of a scary tendency, this fact is easily ascertained by driving a day or two, but it is a most difficult matter to always determine whether or not a horse is sound. The only protection is to secure the services of a good veterinary, and while the result of his investigation is not, in law, absolutely binding on the parties, yet his services always result in a satisfactory finding. Of course, if he should give a certificate of soundness, when the subject of investigation was unsound at the time of his examination, then the vender would still be bound by his warranty, and the purchaser could enforce his contract and compel the vender to return the money and take back his horse, or to sell it at his risk.

1. A warranty is an express or implied statement of something with respect to the article sold, which the seller undertakes shall be a part of a contract of sale; and though part of the contract, yet collateral to the express object of it.

2. It is express where the seller makes some positive representation or affirmation with respect to the article to be sold, pending the treaty of sale, upon which it is intended that the buyer shall rely in making his purchase. An example of an express warranty is had in Wood *vs.* Smith, 4 c. and p. 45, where, upon the sale of a mare, the defendant, knowing the animal to be unsound, remarked to the plaintiff : "I believe the mare to be sound, but I will not warrant her." And in an action for breach of warranty, it was objected by defendant's counsel that the action was wrongly brought in contract, and that it should have been brought in tort on the deceit. But Lord Tenterden ruled that this being a representation made at the time of sale that the mare was sound to the best of the vender's knowledge, was part of the contract; and the plaintiff had a verdict, which the court afterwards refused to disturb. Another illustration is the case of Morrill *vs.* Wallace, in which plaintiff purchased a lot of "lean pork," inquiring at the same time whether it was good, as there appeared to be some unfavorable appearances in it, and defendant stated that it was good, not being at the time aware that it was tainted, as it in fact turned out to be The judge instructed the jury that this constituted a warranty, and a majority of the court on appeals thought a verdict for the plaintiff could be supported, though a new trial was granted, as the judge who tried the case thought, under the circumstances, it should have been left to the jury to find whether, under the evidence given, this was intended as a warranty or expression of opinion only.

3. A warranty is implied where, from the circumstances surrounding the parties at the time of the sale, or from the nature of the thing sold, the law assumes it to be just that the buyer should be protected, in addition to the contract of sale, by a further implied contract or guarantee on the part of the vender, and so raise by implication a warranty on the seller's part. There are several warranties that are implied by law, and their general nature is substantially perceived in the summary by Clayton, J., as follows: In Otts *vs.* Alderson, implied warranties have been ranged under five distinct classes: First, a warranty is implied that a seller has title. Second, that the articles are merchantable when from their nature or situation at the time of the sale an examination is impracticable. This rule is most frequently brought into requisition where the seller is the manufacturer. Third, upon an executory contract to manufacture an article or to furnish it for a particular purpose, a warranty will be implied that it is reasonably fit and proper for such purpose and use, as far as an article of such kind can be. Fourth, a warranty is implied against all latent defects in two cases: (1) Where the seller knew the buyer did not rely on his own judgment, but on that of the seller, who knew or might have known the existence of the defects; and (2) where a manufacturer or producer undertakes to furnish articles of his manufacture or produce in answer to an order. Fifth, that goods sold by sample in quality.

4. Though it is extremely common to find some kind of warranty included in a sale, a warranty does not therefore constitute an essential element of the contract, but the sale is complete without it. In McFarland *vs.* Gibson, Newman, C. J., in speaking of this subject, said: "A sale is a contract executed, on which, of course, no action can be directly founded; but an action may be founded directly on a warranty, and it was doubted in Stuart *vs.* Wilkins, Doug. 18, whether an action could be maintained for a breach of it in any other way; consequently, though it is a concomitant, it is also a collateral, self-existent contract, and no more a part of the sale than a covenant of warranty in a deed is a part of the conveyance;" and in Dorr *vs.* Fisher, Shaw, C. J., remarked that a warranty is a separate, independent, collateral stipulation on the part of the vender, with the vendee, for which the sale is the consideration, for the existence of truth of some fact relating to the thing sold. It is not strictly a condition, for it neither suspends nor defeats the completion of the sale, the vesting of the thing sold in the vendee, nor the right to the purchase money in the vender. And, notwithstanding such warranty or any breach of it, the vendee may hold the goods, and have a remedy for his damages by action." It is true that conditions and warranties, which are often somewhat difficult to distinguish, have been at times confounded, as, for example, the implied conditions precedent of existence, and identity or genuineness in a sale by description, which are always necessarily present in an executory contract of sale, have been called warranties, and warranties thus made essential elements of the sale; but it is very clear, and this will be hereafter spoken of more at length, that these conditions, like all others, are not warranties at all, and that the failure to carefully distinguish the two has caused great confusion in the law.

5. It follows, then, since a warranty is not implied by the mere fact of a sale, but is a self-existent contract and collateral to the sale, that in the absence of an express or implied warranty, the purchaser takes upon himself the risk of the quality of the goods he buys; and this principle of the common law is generally expressed by the maxim of *caveat emptor*. This was early put thus by

Fitzherbert, in the Nature Brevium, at page 213 (seventh edition): "If a man do sell unto another man a horse and warrant him to be sound and good, etc., if the horse be lame or diseased that he cannot walk, he shall have an action upon the case against him. And so if a man bargain and sell unto another certain pipes of wine and warrant them to be good, etc., and they are corrupted, he shall have an action upon the case against him. But it is essential that he warrant it to be good and the horse to be sound, otherwise the action will not lie. For if he sell the wine or horse without such warranty, it is at the other's peril, and his eyes and his taste ought to be his judges in the case (26 H., VI. 35). But if a smith prick my horse with a nail, etc., I shall have my action upon the case against him without any warranty by the smith to do it well; for it is the duty of every artificer to exercise his art rightly and truly as he ought."

In Fenn *vs.* Harrison (1790) it appeared that defendants had directed their agent to cash or discount a bill of exchange in the market, but stated that *they would not endorse it.* And it was held the agent could not bind the defendants to pay the bill, and it was held the agent could not bind the principal by endorsement.

Ashurst, J., on the first trial of the case, made some remarks with respect to the different rules of law applicable where there was a private direction by a master to the case of a general and special agent, saying: "I take the distinction to be that if a person keeping a livery stable and having a horse to sell, directed his servant not to warrant him, and the servant did nevertheless warrant him, still the master would be liable on the warranty, because the servant was acting within the general scope of his authority, and the public cannot be supposed to be cognizant of any private conversation between master and servant.

But if the owner of a horse were to send a stranger to a fair with express directions not to warrant the horse, and the latter acted contrary to orders, the purchaser could only have recourse to the person who actually sold the horse, and the owner would not be liable, because the servant was not acting within the scope of his employment.

In Helyear *vs.* Hawke (1803) a horse was sold by the defendant's groom at Tattersal's, and no warranty was shown in the case, but Lord Ellenborough remarked, "I think the master having intrusted the servant to sell, he is intrusted to do all he can to effect the sale, and if he does not exceed his authority in so doing, he binds his master."

This was followed by the much quoted case of Alexander *vs.* Gibson (1811). There it was decided that a servant sent to sell a horse at a fair was authorized to give a warranty of soundness, because, as Lord Ellenborough said, it is now most usual on the sale of horses to require a warranty, and the agent who is employed to sell when he warrants the horse may fairly be presumed to be acting within the scope of his authority.

Then came the well-known case of Brady *vs.* Todd, containing the able statement of law on this point. Here the defendant, a potato salesman in London, owning a farm in Essex, which was under the care of a bailiff, had recently purchased a horse and sent it to his farm.

A friend of the plaintiff, on the lookout for a horse for him, hearing of this, appealed to the defendant for the purchase, whereupon the bailiff was sent with the horse to the plaintiff and authorized to sell the animal for not less than thirty guineas. The jury found that no express authority had been given to the bailiff to make a warranty, but that the latter had warranted the horse. On the argument the counsel for the defendant insisted that no authority to warrant could be implied from the authority to sell.

But the Judge appeared to have made a distinction between horse dealers and others.

If Tattersal sent his servant to sell, and the servant, contrary to his instructions, warranted, Tattersal might be bound, but not another person (not a horse dealer) would not be bound by the unauthorized warranty. Judge Erle, C. J., in delivering the opinion, observed that the point had never been decided, though often mentioned by Judges and text writers. The general rule that the act of an agent does not bind his principal unless it is within the authority given him, is clear. But the plaintiff, that the circumstances created an authority in the agent to warrant on various grounds.

Among others he referred to cases where the agent has by law a general authority to bind his principal, though as between themselves there was no such authority such as partners, and stress was laid on the expression of several judges that the servant of a horse dealer or livery stable keeper can bind his master by a warranty, though as between themselves there was an order not to warrant. We understand these Judges to refer to a general agent employed by a principal to carry on his business—that is, the business of horse dealing. In which case there would be by law the authority contended for. But the facts of the present case do not bring the defendant within this rule, as he was not shown to carry on any trade of dealing in horses.

It is also contended that a special agent, without any express authority in fact, might have an authority by law to bind his principal!

The main reliance was on the argument that an authority to sell is by implication an authority to do all that in the usual course of selling is required to complete the sale, and that the question of warranty is in the usual course of a sale required to be answered, and that therefore the defendant by implication gave the bailiff authority to answer that question and to bind him by his answer. It was a part of the argument that the agent authorized to sell a horse had authority to warrant, but on this point the plaintiff has in our judgment failed. We are aware that the question of warranty frequently arises upon the the sale of horses, but we are also aware that sales may be made without any warranty or even an inquiry about warranty.

If we laid down for the first time that the servant of a private owner intrusted to sell and deliver a horse on one particular occasion is therefore by law authorized to bind his master by a warranty, we should establish a precedent of dangerous consequences. For the liability created by warranty extending to unknown as well as known defects is greater than is expected by persons unexperienced in law. We therefore hold that the buyer taking a warranty from such an agent as was employed in this case takes it at the risk of being able to prove that he had the principal's authority, and if there was no authority in fact the law does not from the circumstances in our opinion raise one.

The following defects have been considered to constitute unsoundness in horses :

The glanders ; "corns ;" bone spavin in the hock ; the navicular disease ; any organic defect ; blindness or defect in one eye ; ossification of the cartilages ; thick wind ; whether a splint would constitute unsoundness would depend upon its situation ; roaring ; crib-biting, affecting the health ; mere badness of shape, which has not produced lameness at time of sale, is not unsoundness ; a convexity in the eye causing near-sightedness, and bad habits therefrom, is unsoundness.

There has been considerable variance in the decisions as to what words or expressions constitute an express warranty, and while it is now generally conceded that there is no set form of words required to constitute an express warranty, yet not every positive averment, or even express representation, is deemed to be an express warranty.

Perhaps the earliest decision in this State on the law of warranty in the sale of a horse, was in the case of Jackson *vs.* Wetherill, 7 S. & R. 480, where the seller assured the purchaser that the mare, which was the subject of the sale, was perfectly safe, kind, and gentle in harness, and the court held that the words used did not amount to an express promise or engagement that she was so, much less to a direct warranty. In McFarland *vs.* Newman, 9 Watts, 55, Chief Justice Gibson said: "Though to constitute a warranty requires no particular form of words, the naked averment of a fact is neither a warranty of itself nor evidence of it. In connection with other circumstances, it certainly may be taken into consideration; but the jury must be satisfied from the whole that the vender actually, and not constructively, consented to be bound for the truth of his representation. Should he have used expressions fairly importing a willingness to be thus bound, it would furnish a reason to infer that he had intentionally induced the vendee to treat on that basis; but a naked affirmation is not to be dealt with as a warranty, merely because the vendee had gratuitously relied on it, for not to have exacted a direct engagement had he desired to buy on the vender's judgment, must be accounted an instance of folly. Testing the vender's responsibility by these principles, justice will be done without driving him into the toils of an imaginary contract.

The latest case is Holms *vs.* Tyson, Atlantic Reporter, Vol. XXIII, p. 564. In that case, at the time the transaction was closed and the money paid, there was no warranty. On the contrary, the plaintiff said to the defendant: "I have nothing to show that you warrant this horse as you represent him;" to which the defendant replied: "The horse is just the same as when you drove him on Monday." It was held by the court: "This is very far from being a warranty. It was, at most, an assertion that the horse was in the same condition as on the previous Monday, and there was nothing in the case to show that it was not true. There was evidence of previous statements having been made to the plaintiff that the horse was kind, sound, and gentle, but the defendant did not warrant him to be so." From these decisions we gather the law to be, that to constitute a verbal express warranty there must be such words used at the time of the sale as would clearly lead the purchaser to believe that he had a right to rely on them as warranting the soundness and character of the horse, and at the same time must be of such a certain nature and intendment as to show that the seller meant to be bound by what he said.

When a horse is sold on a verbal warranty the law does not fix any time when the warranty is to cease, but it must be a reasonable time, such a time as would give an ordinarily careful man an opportunity to ascertain whether or not the guarantee is correct, usually a few days. But it is the duty of a purchaser as soon as he discovers that a horse is not as warranted to immediately notify the vender of the fact, and if he desires the contract rescinded he must at once return the horse and demand back the purchase money, and if the vender refuses to do this, it is then his duty to sell the horse at public sale, giving the vender notice of time and place, and if he sustains a loss his remedy is then an action at law against the vender for the difference. But if the purchaser exercises the rights of ownership after he discovered a breach of warranty, he could still have his right of action and keep the horse, and the amount of his damage would be the difference in value between the horse as represented and as he is actually found to be. This is often a difficult matter to prove, and that is the reason the custom has been to sell the horse at public sale where the vender refuses to take him back and refund the money ; but while this is the better practice, yet a man may keep the horse and still have his right of action.

The warranty of soundness, of course, gives the greatest amount of contention and dispute, and such will be the case as long as time lasts, for it is impossible to get doctors to agree, and it is a very difficult thing to get half a dozen veterinarians who will agree about the condition of a horse as to soundness. I think I can safely say that I have never yet seen a case tried in our courts in which testimony of veterinary surgeons has not been directly opposite ; so long as this is possible, so long will people litigate about horses ; when the science of surgery becomes so simple (which will never be), then, and only then, will disputes and litigations end.

And I know of no profession that requires greater skill and study than that required by the veterinary. If you call in a physician to attend a member of your family, as a general rule he can intelligently diagnose the case.

The patient (unless an infant) can tell him where the disorder is located, how long it has existed, just the kind and nature of the pain, and give in detail a dozen other facts and incidents that are of the utmost advantage in aiding the physician in ascertaining the nature and cause of the disease, and yet with all these advantages how often does the physician fail to ascertain the the true illness, or to give the proper treatment! How much more difficult is it where doctor has to confront a poor dumb animal that cannot impart to him the location of the disease or any of the facts that might enable him to arrive at a satisfactory conclusion. His diagnosis must be imperfect, as he must take his facts from the best means possible, which are a careful examination; after failing to discover the real source of illness, sometimes an inward pain or an inward unsoundness, of which he has no means of ascertaining the real cause of. With all these difficulties, it is not to be wondered at that horse doctors do not agree; and when we consider the great number of diseases that the horse is heir to, we can hardly expect to improve very much over our present knowledge.

Here a word about the relation that exists between the veterinary surgeon and the person who employs him. The relations are precisely the same as that of the family physician. He is liable in damages for unskillful or negligent treatment, nor can he recover for his services when they have been negligent or unskillful; of course this can only be determined, as a general rule, by the statement or testimony of others in his profession, as that when our horse has been improperly and unskillfully treated we must call in another veterinary to discover that fact.

It has grown to be almost a universal practice to give a certificate as to soundness or unsoundness where the surgeon is called in. This certificate is binding on the veterinary, and if he gives a certificate which is untrue, he holds himself liable in damages, because it is upon his certificate that a large sum of money is often paid out, and as he is paid for his opinion, the law presumes that he is competent to give a correct one. Of course the law does not expect any one to do impossibilities, and where a veterinary gives a certificate of soundness, and it afterwards turns out that the horse is unsound, but the nature of unsoundness is of such a character that it is beyond human possibility to ascertain it, he is relieved from responsibility.

Sometimes it occurs that in the sale of a horse two parties agree to refer the question of soundness to a certain veterinary whom they both know, and in such a case his action is final and binding on both parties, and neither one can afterwards rescind the contract or maintain an action for breach of warranty.

There is another species of trading and selling horses which seems to be of modern origin, and seems for the most part to be a sort of specialty, or in other words to be engaged in by a class of people who have no regard to their character or reputation. I mean that class of horse dealers who are known as "Gyps." These people would be "Gyps" in any business. If they were in the clothing business they would be located on South street with barker in front of their store, and would ask $40 for a suit of clothes that they would sell for $4. The only difference is they have adopted selling horses instead of clothing for the purpose of making a dishonorable and dishonest livelihood, and they should receive no encouragement or consideration either at the hands of the public, the reputable veterinary, or the reputable horse dealer.

It should be the object and aim of the reputable dealers to drive them out of the business, and I have often thought it would be a laudable undertaking on the part of the better class of horse dealers to form a combination or an association for the purpose of preventing them from carrying on their infamous business. These people generally catch the unwary and unsuspecting purchaser by means of an advertisement, and they are all well known to the legitimate dealer. How easy it would be to notify the public under their advertisement, and in the same paper, John Jones, who advertised a horse for sale at No. —, is not a legitimate dealer, but what is known as a "Gyp," and no man should purchase from him. And then they have their envoys at all public sales, and whenever they find a man in search of a horse they approach him to introduce themselves and tell him they have a friend who has just the horse he wants, and the unsuspecting purchaser is ushered into some "Gyp's" stable, and often made the object of a bare-faced swindle. Why should not these people be driven from such places and not allowed to attend public sales by the people who conduct such sales?

This class of dealers have introduced into our criminal courts a new class of cases—that of obtaining money under false pretenses, and while these cases are of a frequent occurrence in our criminal courts, they are of recent origin. I account for that in two ways: First, because this mode of horse dealing is of recent origin; and second, because there was some doubt of the act constituting a crime. I think I can safely make the assertion that prior to ten years ago no man was ever tried in our criminal courts for the offense of receiving money under false pretences in the sale of a horse, and while it seems to be the only way of reaching this class of dealers, yet I have serious doubts whether the act of selling an unsound horse, representing it to be sound, constitutes the offense under the law of obtaining money under false pretenses, although judges more learned in the law than I am have sustained convictions of this offense and have sentenced the offenders to imprisonment, although in most cases the offenders get off by making restitution and paying all expenses arising out of the prosecution. I do not think any of these convictions have ever been ratified by the Supreme Court, because I know of no case that has been taken to the Supreme Court. Of course to sustain a case of this kind it is necessary to satisfy the court and jury that the representations made at the time of the sale were false, and that the person selling the horse and making the representations knew that they were false at the time he made them ; all of these cases arise out of the sale of horses that are unsound, and the cases are made out and convictions secured in one of two ways : First, by proving as an actual fact that the seller knew that the animal was unsound, by producing a witness who can testify to the fact that he saw the horse in the possession of the seller at the time when the disease or unsoundness was visible, or when it was the subject of conversation and comment.

The other is by producing a veterinary surgeon or expert who has examined the horse and who can testify, from the nature of the disease or unsoundness, it must of necessity have been known to the owner who made the sale before or at the time he sold it. Of course, in order to secure a conviction it is necessary to prove that the horse was unsound, and that the owner knew that fact at the time he sold it.

There are many instances in which a man sells an unsound or diseased horse and does not know the fact at the time of the sale. To this class of people the criminal law does not apply, and the only remedy is a civil action for breach of warranty. The reason that the courts recognize such transactions as a criminal offense, in my opinion, is that the class of people who generally engage in such transactions are generally irresponsible, and a verdict in the civil courts would be of no benefit to the injured purchaser, as he could not recover on his verdict.

There is another method of punishment that the courts sometimes enforce, and that is, where two or more men, knowing a horse to be unsound, enter into a combination or agreement to deceive a purchaser by representing the horse as sound. This is a conspiracy at common law, and all parties participating knowingly in such a sale are guilty of a crime, whether they be owners or merely assisting the owner. In all this class of cases the only protection to the purchaser is to retain the services of a good veterinary; and no man should purchase a horse of any value, unless he is an expert, without first getting the judgment of a good veterinary, which is always cheaper in the end.

There is custom among many persons, who have more money than discretion, to take the judgment of his coachman in such transactions, and he, often incompetent to judge, and perhaps oftener becomes a party to the fraud, by shutting his eyes to any defects and aiding in the perpetration of a fraud for a proportional part of the purchase money or bribe. And you gentlemen know how hard it is to sell a horse, or pair, to a man of means, until you have first satisfied the coachman by a substantial payment, that the horses are about what his master wants and needs. This is a practice that should be abolished ; but I am free to say that the competition is so great and the practice of such long standing, that I am unable to give any remedy. Of course, if an association of legitimate horse dealers was formed and existed, just as other associations exist, this evil might be remedied, but not until then ; but as the seller, under such circumstances, gets a good price for his horse, the temptation is very strong and the habit very likely to continue.

Where a veterinary surgeon is called in to give his opinion about a horse, he should be very careful about the character of certificate he gives, for I believe it is a conceded fact that there are certain defects and diseases that are beyond the comprehension and detection by the most learned upon a casual examination, and in order to protect the veterinarian, it is proper that you except these defects or diseases in his certificate. There are few men who call in a veterinary to make an examination and get his honest judgment, after a careful examination, who would undertake to hold him responsible for an error, either of judgment or skill, but now and then you find a man who will not suffer a loss without trying to make some one sustain it.

